# Help-Seeking Behavior and Treatment Barriers in Anxiety Disorders: Results from a Representative German Community Survey

**DOI:** 10.1007/s10597-020-00767-5

**Published:** 2021-01-20

**Authors:** Ingmar Heinig, Hans-Ulrich Wittchen, Susanne Knappe

**Affiliations:** 1 Institute of Clinical Psychology and Psychotherapy, Technische Universität Dresden, Chemnitzer Str. 46, 01187 Dresden, Germany; 2 Department of Psychiatry and Psychotherapy, Ludwig-Maximilians-Universität München, Nußbaumstr. 7, 80336 Munich, Germany

**Keywords:** Anxiety disorders, Service use, Help seeking, Treatment barriers, Dropout

## Abstract

Although effective therapies exist, treatment rates of anxiety disorders (AD) are low, raising the question why affected individuals do not receive treatment. We provide data from the nationally representative German Health Interview and Examination Survey-2011 (DEGS1) on the help-seeking behavior and perceived treatment barriers of 650 subjects with Diagnostic and Statistical Manual of Mental Disorders’ (DSM-IV AD). Only 26% of all cases with AD in the community reported having had contact with mental health services because of their anxiety problems in their lifetime. 16% were currently receiving professional help, most frequently by psychotherapists (8%), psychiatrists (5%) and general practitioners (5%). 40% of all cases never even considered seeking help and 31% reported barriers to treatment, such as self-reliance (18%) or beliefs that treatments were ineffective (9%), unavailable (8%) or too stigmatizing (7%). Measures to increase treatment rates should thus target individual as well as public attitudes and health literacy to increase awareness of and access to evidence-based interventions.

**Supplementary information:**

The online version of this article (10.1007/s10597-020-00767-5) contains supplementary material, which is available to authorized users.

## Introduction

Anxiety disorders (AD) are the most prevalent group of mental disorders and are responsible for high levels of individual and societal disease burden (Alonso et al. 2004a; Jacobi et al. 2014; Kessler et al. 2012; Wittchen and Jacobi 2005; Wittchen et al. 2011). For example, individuals with AD report 5.9 days with functional impairment per month (Mack et al. 2015) and societal costs are estimated at more than 1.000€ per case and year in Europe (Gustavsson et al. 2011). AD are successfully treatable with evidence-based psychological and pharmacological interventions, such as cognitive-behavioral treatments (Bandelow et al. 2014; Butler et al. 2006; Carpenter et al. 2018; Norton and Price 2007). However, as demonstrated by numerous studies since the 1980s, the majority of affected individuals do not receive professional help (Essau 2005; Jacobi et al. 2004; Kessler et al. 1994; Magee et al. 1996; Runge et al. 2008; Wang et al. 2007; Wittchen et al. 1999). It is reported that only around half of all cases have ever contacted mental health services, and about one quarter have seen a specialized professional (Alonso et al. 2004b; Bijl and Ravelli 2000; Wittchen et al. 2011). Even worse, there is an average delay of six years between onset and first service use (Mack et al. 2014). Service use in AD has apparently not been increasing significantly during the last three decades (De Graaf et al. 2012; Wang et al. 2005). This may indicate that relevant treatment barriers have not been sufficiently targeted throughout this time (Mack et al. 2014). The present study will thus examine characteristics of help-seeking across different AD, as well as putative reasons not to seek treatment.

Service use, help-seeking, disorder-specific treatment and treatment completion can all be seen as sequential steps of health care utilization (note that while help-seeking can also occur at an informal level, i.e. support from friends, spiritual care etc., in this study we exclusively refer to formal help-seeking). *Service use* refers to any contact with mental health-care providers, irrespective of its purpose (Alonso et al. 2004b; Wang et al. 2007; Wittchen and Jacobi 2001). *Disorder-specific help-seeking* denotes that an individual addresses their anxiety symptoms if in contact with a mental health-care provider (Mackenzie et al. 2012). Further, a disorder-specific *treatment* is present if the concerned individual has repeated contacts with a specialized professional and disorder-specific interventions are applied (Fernández et al. 2007; Wang et al. 2007).

To date, extensive data on general service use in AD is available (Alonso et al. 2004b; Mack et al. 2014; Wang et al. 2005). However, service use rates do not fully reflect care and treatment of AD: A substantial fraction of individuals with AD are in contact with help services but present with other problems such as somatic or depressive symptoms (Asp et al. 2020; Kirmayer et al. 1993; Zimmerman and Chelminski 2003). It is reported that less than half of the potential generalized and social anxiety diagnoses are detected in primary and outpatient psychosomatic services (Wiltink et al. 2010; Wittchen et al. 2002). Consequently, only 30%–70% of service users with AD receive an anxiety-specific treatment (Fernández et al. 2007; Roberge et al. 2011; Stein et al. 2004). Moreover, help-seeking rates seem to differ between the various types of AD. For example, individuals with panic or generalized anxiety disorder (GAD) are reportedly high utilizers of treatment services (Wittchen et al. 2003), whereas individuals with specific phobias (SPEC) rarely seek any treatment (Mackenzie et al. 2012). Treatment barriers might also differ between AD (Olfson et al. 2000), but disorder-specific barrier profiles have rarely been reported. The present study thus focusses on *disorder-specific* help-seeking and its barriers.

Help-seeking for mental health problems is associated with *sociodemographic* factors such as age, gender, and urbanization, with *need* factors such as disorder severity, comorbidity, or impairment, and with *enabling* factors such as knowledge on mental health services (Goodwin and Andersen 2002). Barriers to help-seeking may be deliberately *perceived*, i.e. an individual faces specific obstacles when trying to seek help, or *unperceived*, i.e. an individual does not even consider seeking help and therefore does not experience any barrier. Typical perceived barriers in individuals with AD are knowledge deficits (e.g. knowing where he/she can seek help), fear of stigma and reliance on self-management strategies (Clark et al. 2018; Coles and Coleman 2010; Mojtabai et al. 2011).

Even when individuals with AD enroll in treatment, they do not necessarily receive adequate therapy in terms of type or dosage, nor do they always complete a treatment. They may drop out of therapy (Fernandez et al. 2015; Taylor et al. 2012) prematurely, depending for example on attitudes and expectations towards treatment (Bélanger et al. 2017; Santana and Fontenelle 2011). Treatment dropout is typically associated with increased economic costs of the illness (Konnopka et al. 2009; Wittchen et al. 2011). The assessment of reasons for dropout is therefore an important supplement to overcome treatment barriers.

In sum, although low service use has been described for AD, evidence on disorder-specific help-seeking behavior and on correlates and predictors of service utilization is sparse. We therefore aim to report (1) disorder-specific help-seeking rates for different health sectors and providers, (2) information on the extent and type of treatment with the most frequently utilized specialized services, (3) predictors of help-seeking, and (4) information on treatment barriers and dropout reasons in AD.

## Methods

### Sampling

Data for this study stem from the German Health Interview and Examination Survey for Adults—Mental Health module (DEGS-MH, 2011), a nationally representative survey designed to examine prevalence, impairment, and help-seeking for mental disorders in the German adult population (Jacobi et al. 2013, 2014). DEGS-MH is a module of the German Health Interview and Examination survey (DEGS1, Scheidt-Nave et al. 2012). DEGS1 used a two-stage stratified cluster sample (N = 8152), based on (1) 180 sample points across Germany, stratified for grade of urbanization, population density and administrative borders (2) random selection of participants from local population registries at each sample point (Kamtsiuris et al. 2013). Participants of the previous German health survey, dating from 1998, were also included. DEGS1 participants were eligible for DEGS-MH if they fell into the specified age range (18–79 years), had fully completed DEGS1, had given informed consent for DEGS-MH and were available for the survey. Of 6028 eligible individuals, 4483 (74.4%) completed DEGS-MH. 8.5% were excluded because they refused to participate, 3.3% could not be contacted, and 13.8% took part in a reduced core survey. Assessments were conducted by clinically trained assessors at all 180 sample points by way of computer-assisted personal interviews between 09/2009 and 03/2012 (for details see Jacobi et al. 2013). The present study used the subsample of 650 cases who met criteria for a 12-month AD according to DSM-IV-TR. Sample characteristics are displayed in Table 1.

**Table 1:** Sample characteristics

	N	Any AD		PD		AG		SAD		GAD		SPEC	
		650		93		164		95		87		423	
Demographics													
Age	M (SD)	45.2	(15.7)	45.7	(12.0)	46.5	(15.7)	39.6	(12.9)	43.8	(15.1)	46.1	(16.3)
Female sex	% (N)	70.4	(458)	71.3	(70)	71.5	(113)	67.6	(57)	66.5	(55)	75.5	(317)
Employed	% (N)	56.8	(362)	57.9	(55)	50.9	(83)	44.5	(48)	51.4	(44)	54.9	(232)
Living with a partner	% (N)	70.4	(460)	66.6	(58)	64.3	(103)	64.3	(64)	67.3	(64)	71.8	(302)
Clinical characteristics													
Age of onset	M (SD)	36.7	(18.6)	31.1	(14.8)	30.3	(17.3)	20.2	(13.2)	32.9	(15.7)	40.4	(18.9)
Duration (years)	M (SD)	8.5	(13.6)	14.6	(13.3)	16.2	(15.5)	19.4	(16.0)	10.9	(14.0)	5.7	(12.4)
Disability (y/n)	% (N)	40.0	(232)	64.8	(58)	49.1	(83)	71.3	(66)	60.6	(46)	34.2	(127)
No. of comorbidities	M (SD)	1.4	(1.6)	2.0	(2.0)	1.9	(2.0)	2.7	(2.4)	1.9	(1.9)	1.4	(1.7)
Comorbid AD (y/n)	% (N)	27.0	(155)	67.1	(59)	65.5	(102)	59.8	(58)	44.7	(34)	32.2	(114)
Comorbid DD(y/n)	% (N)	27.7	(169)	32.7	(33)	33.8	(56)	43.4	(39)	54.3	(46)	24.3	(86)

AD = anxiety disorder, PD = panic disorder, AG = agoraphobia, SAD = social anxiety disorder, GAD = generalized anxiety disorder, SPEC = specific phobia, DD = depressive disorder

### Measures

#### Anxiety Disorders

AD included in this study were panic disorder (PD), agoraphobia (AG), social anxiety disorder (SAD), GAD and SPEC. Diagnoses were assessed using the DIA-X software, a computerized German version of the Composite International Diagnostic Interview (CIDI; Wittchen and Pfister 1997). The CIDI is a fully standardized interview with good reliability and validity for AD (Reed et al. 1998; Wittchen 1994; Wittchen et al. 1998). Diagnoses were assessed according to the Diagnostic and Statistical Manual of Mental Disorders’ (DSM-IV-TR) criteria during the past 12 months.

#### Service Use

Service use was estimated as a condition for help-seeking (see below). The CIDI included a section on service use behaviors and disability, placed at the end of the interview. Participants were first asked whether they had ever thought about or had been recommended professional help due to mental health or psychosomatic problems. They were then presented a list with 27 services to indicate which of them they had ever contacted because of mental health problems. The list of services included primary care (i.e., general practitioners), inpatient (e.g., psychiatric or psychosomatic hospitals) and outpatient institutions (e.g., psychiatrists, outpatient clinics), as well as complementary institutions (e.g., counselling centers, helplines; full list available upon request). For each institution, in-depth questions were asked to explore occasion, duration and type of intervention. This information was used to calculate 12-month vs. lifetime help-seeking rates.

#### Disorder-Specific Help-Seeking

Participants were considered as help-seekers if they had (1) used at least one health service from the list of health care services and (2) had at least once talked to a professional about their anxiety symptoms. The latter information was included in the CIDI’s diagnostic section for each AD (*“Have you ever talked to a professional, e.g. medical doctor, psychologist, social worker, counsellor, clergy, about these problems?”*). For SPEC, the question: *“For what reason did you visit or contact these institutions?”,* asked in the CIDI’s service use section, was used to indicate disorder-specific help-seeking.

#### Disability

Current disability due to mental disorders was assessed as a potential predictor of help-seeking at the very end of the interview by asking participants whether they had been “at least partly impaired in their everyday activities (work, household, etc.) due to mental or psychological problems” at any day within the past four weeks.

#### Treatment Barriers and Reasons for Dropout

The CIDI’s service use section also included gated questions regarding treatment barriers and potential reasons for dropout. Those who agreed to have ever thought about seeking professional help (see above) were then asked for treatment barriers using a list of 13 possible barriers (yes/no): *“Has it ever happened that you thought about or have been recommended professional help, but then did not do it? Why was that?”* Participants who never thought about seeking help were not asked for barriers and are here reported as individuals with “unperceived barriers”. Participants who reported to have discontinued at least one treatment were asked for dropout reasons using a list of 16 possible dropout reasons (yes/no). Item lists for services, barriers and dropout reasons are available in the supplement.

### Statistical Analysis

Statistical analyses were conducted using Stata 14.2 (StataCorp 2017). To ensure representativity for the German population, post stratification weights based on 11 demographic, socio-economic and geographic variables (e.g., age, nationality, region) and the probability to participate in the DEGS-MH module were applied in all analyses and weighted percentages, means, standard deviations and confidence intervals (CIs) are reported. A description of the weighting procedure is given by Jacobi et al. (2013). To test potential predictors of help-seeking, weighted logistic regression was used and odds ratios (OR) are reported. Tests were two-sided with *α* = 0.05.

## Results

### Prevalence and Type of Disorder-Specific Help-Seeking

#### Lifetime Help-Seeking

Among all cases with a 12-month AD, 26.0% reported lifetime use of mental health services due to their disorder. Rates ranged from 9.5% in SPEC to 67.3% in PD (see Table 2). The most frequently used outpatient services were psychological and medical psychotherapists (16.7%), psychiatrists (9.2%), and GPs (8.1%). 5.1% contacted other outpatient services, including outpatient clinics (3.1%), other psychologists (2.2%), social-psychiatric services (0.7%), and others (1.1%). A remarkable 11.2% reported inpatient service use like psychosomatic hospitals (6.6%), psychiatric hospitals (3.8%), or daycare clinics (3.0%; other 1.7%). 6.4% reported using what is labeled as “complementary services” (counseling centers, 5.6%, self-help groups, 0.3%, other 1.5%) for their AD symptoms. The ranking of most frequently used services was equal across all AD. On average, help-seekers reported to have consulted 2.5 (*SD* = 1.7) different services in their lifetime. The number of services contacted was highest in SAD (3.2, *SD* = 1.8) and lowest in GAD (1.9, *SD* = 1.7).

**Table 2:** Disorder-specific help-seeking in AD

	Any AD		PD		AG		SAD		GAD		SPEC	
	N	%	N	%	N	%	N	%	N	%	N	%
Lifetime help-seeking	155	26.0	59	67.3	56	36.9	51	53.3	39	50.9	26	9.5
Primary care	58	8.1	24	28.9	24	13.2	25	22.7	10	9.5	13	3.9
Outpatient services	129	22.0	52	61.3	49	30.2	46	50.1	28	39.3	24	8.2
Psychotherapist	89	16.7	37	45.5	34	24.1	37	43.4	18	26.7	17	6.1
Psychiatrist	63	9.2	30	33.4	30	18.1	24	25.6	8	7.9	10	3.0
Other	32	5.1	9	12.3	13	7.7	14	12.2	7	8.5	8	2.8
Inpatient services	67	11.2	32	37.0	30	20.3	26	24.3	14	12.0	10	4.0
Complementary services	31	6.4	9	14.4	14	10.1	13	17.9	9	14.3	5	2.2
12-month help-seeking	92	15.9	36	41.8	34	22.6	35	37.5	29	40.4	12	3.9
Primary care	33	4.6	15	16.6	10	5.6	12	9.5	8	6.5	6	2.1
Outpatient services	69	12.4	29	35.3	30	19.2	29	32.5	17	26.7	12	3.9
Psychotherapist	38	7.9	18	21.3	17	14.1	17	23.3	9	15.4	7	2.0
Psychiatrist	30	5.1	13	18.3	15	10.9	13	15.2	4	4.9	5	1.9
Other	12	2.4	5	7.7	5	3.3	5	5.0	4	6.5	3	1.1
Inpatient services	13	1.8	7	7.8	7	3.3	6	4.1	5	4.6	1	0.1
Complementary services	10	3.2	3	6.8	5	4.9	4	7.7	3	9.8	2	0.6

AD = anxiety disorder, PD = panic disorder, AG = agoraphobia, SAD = social anxiety disorder, GAD = generalized anxiety disorder, SPEC = specific phobia

#### 12-Month Help-Seeking

In the past year, 15.9% of all cases with 12-month AD contacted mental health services to manage their disorder, ranging from 3.9% in SPEC to 41.8% in PD (see Table 2). The most frequently used services were psychotherapists (7.9%), psychiatrists (5.1%) and GPs (4.6%). 2.4% contacted other outpatient services (outpatient clinics 1.4%, other psychologists 0.7%, social-psychiatric services 0.3%, others 0.4%). 1.8% used inpatient services (psychosomatic clinics 0.7%, psychiatric hospitals 0.4%, daycare clinics 0.2%, neurological clinics 0.2%, other 0.3%), and 3.2% used complementary services (counseling centers 2.7%, self-help groups 0.1%, other 0.4%) for AD symptoms. The ranking of most frequently used services was different in GAD, were counselling centers were contacted relatively more often. 46.8% of help-seekers had used more than one service in the last 12 months (*M* = 1.6, *SD* = 0.8).

SPEC constituted the largest diagnostic group in the sample and, at the same time, showed the lowest help-seeking rates. Since SPEC are also characterized by lower impairment in daily life and lower perceived need for help compared to other AD (Mack et al. 2015 also see severity indicators in Table 1), they might negatively bias our interpretation of help-seeking rates. We therefore additionally calculated help-seeking rates (Table 2, column 1) without SPEC. This resulted in an overall 12-month help-seeking rate of 20.0% (primary care 5.7%, outpatient services 15.6%, inpatient services 2.3%, complementary services 4.1%).

### Factors Associated with Help-Seeking

In univariate regressions using the complete sample (N = 650), predisposing and enabling factors (age, current partnership, employment and urbanization), as well as all need factors were associated with help-seeking (see Table 3). As anxiety comorbidity showed very large CIs, we repeated the analysis using robust estimation with the Huber-White sandwich matrix. Here, urbanization no longer predicted help-seeking (*OR* = 0.86, *CI* = 0.48–1.57 for 100–500 T inhabitants) and higher education emerged as a predictor (*OR* = 0.44, *CI* = 0.20–0.97). The OR for more three or more comorbid AD was 13.22 (*CI* = 6.71–26.06). In a multivariate regression including all variables, only age ≥65 years (*OR* = 0.13, *CI* = 0.04–0.44) and the need factors “anxiety comorbidity” (*OR* = 8.78, *CI* = 3.05–25.26 for three AD compared to only one AD) and “presence of disability” (*OR* = 4.91, *CI* = 2.33–10.35) remained as predictors.

**Table 3:** Predictors of 12-month help-seeking in individuals with AD

	N	Proportion of help-seekers^a^	Association of predictors with help-seeking			
		%	OR	CI 95%		p
Sociodemographic factors						
Gender						
Women	453	16.9	Ref.			
Men	190	14.1	0.83	0.44	1.54	0.553
Age						
18–34	146	21.1	1.33	0.64	2.77	0.442
35–49	178	16.3	0.99	0.50	1.94	0.967
50–64	196	16.6	Ref.			
65–79	123	4.5	**0.24**	0.07	0.76	0.015
Current partnership						
Yes	454	13.1	Ref.			
No	189	23.1	**1.93**	1.01	3.72	0.048
Educational level						
Low	190	16.7	1.28	0.64	2.56	0.488
Medium	345	17.0	Ref.			
High	106	9.8	0.58	0.22	1.48	0.251
SES						
Low	118	16.6	0.93	0.44	1.95	0.849
Medium	392	17.2	Ref.			
High	131	10.8	0.56	0.26	1.20	0.135
Enabling factors						
Employment						
Yes	360	12.7	Ref.			
No	283	20.6	**2.62**	1.41	4.85	0.002
Urbanization						
<20 T inhibitants	108	17.8	0.93	0.41	2.15	0.872
20–100 T	142	19.1	1.06	0.49	2.29	0.882
100–500 T	183	9.9	**0.46**	0.22	0.97	0.040
>500 T	210	18.9	Ref.			
Need factors						
Disability						
No	418	5.4	Ref.			
Yes	225	32.7	**8.11**	4.35	15.10	0.000
Chronicity						
0–5 years	418	10.9	Ref.			
6–20 years	116	24.8	**2.60**	1.25	5.42	0.011
>20 years	109	25.6	**3.60**	1.77	7.32	0.000
Number of AD						
1	492	8.5	Ref.			
2	105	23.6	**3.49**	1.71	7.13	0.001
3+	46	67.89	**23.70**	10.39	54.05	0.000
Comorbid depression						
No	491	11.3	Ref.			
Yes	152	30.2	**3.32**	1.81	6.12	0.000

Predictors were entered separately into the logistic regression model. All analyses adjusted for sex and age group. Bold values indicate significant ORs

SES = socio-economic status, AD = anxiety disorder, OR = odds ratio, CI = confidence interval, p = p-value

^a^Among individuals with given predictors

### Characteristics of Specialized Treatments

Of those cases with lifetime help-seeking with a psychotherapist or psychiatrist, 92 (79.3%) answered more detailed questions on treatment characteristics (see Table 4).

**Table 4:** Characteristics of lifetime specialized outpatient treatments

	Psychiatrist (N = 59)		Psychotherapist (N = 55)	
	N	%	N	%
Number of sessions				
1–5	21	38.1	8	21.4
6–30	19	28.7	16	16.4
31–59	7	13.6	12	24.1
≥60	9	14.4	17	34.2
Duration of sessions				
1–10 min	11	18.7	1	1.1
11–30 min	35	61.7	3	11.9
31–49 min	6	6.8	5	5.6
50 min	6	10.8	45	80.3
Duration of therapy				
Up to one year	18	25.8	16	27.2
1–3 years	8	13.9	20	41.2
>3 years	33	60.3	13	31.6
Type of treatment				
Medication	43	76.9	6	12.8
CBT	2	2.6	20	29.7
Other psychotherapy	36	56.2	37	70.1
Group therapy	0	0.0	3	2.1
Other treatment	8	10.6	3	5.6
Satisfaction with therapy				
(Rather) satisfied	26	42.8	31	56.3
Partly satisfied	23	43.0	18	35.4
Dissatisfied	10	14.2	6	8.3

CBT = cognitive behavioral therapy

Treatment sessions with *psychiatrists* were typically shorter than 30 min (80.4% of cases). Psychiatrists predominantly delivered psychopharmacological interventions (76.9%) or some type of individual psychotherapy (56.2%). Cognitive behavioral therapy (CBT) was rarely mentioned (2.6%), despite being a first line treatment. The average time-lapse from first treatment contact to the last was 11.3 years (*SD* = 15.9, *M* = 5.0), with an average of 5.6 sessions per year (*SD* = 5.9, *M* = 4.0, values calculated for therapies that had been ongoing for at least one year), and a total number of about 30 sessions. On average, treatment by psychiatrists may thus be described as long-lasting treatment with rare contact.

Treatment by *psychotherapists* was delivered as a short-term treatment in about 40% of cases (i.e., up to 30 sessions) and as a long-term treatment in 60% of cases (30+ sessions; note that individuals summed up sessions if they underwent more than one therapy). Duration of psychotherapy sessions was usually 50 min, as prescribed by reimbursement rules in Germany. 29.7% of participants with psychotherapy reported specifically having received some form of CBT, 70.1% any other type of psychotherapy. On average, patients reported having been in psychotherapy for 4.2 years (*SD* = 5.8, *M* = 2.0) and received 23.2 sessions per year (*SD* = 20.8, *M* = 15.3).

In both groups (psychiatrists and psychotherapists), about 50% of patients reported being fully or largely satisfied with their psychotherapeutic or psychiatric therapies. Dissatisfaction with treatment was associated with higher probability of dropout (*OR* = 1.70, CI = 1.20–2.41).

### Treatment Barriers

31.3% (N = 187) of all cases reported barriers to treatment (Table 5). Another 39.9% (N = 285) never considered seeking help, pointing to unperceived barriers. The most frequently reported barriers were self-reliance (“wanted to deal with the problem alone”, 18.4%), perceived ineffectiveness of treatment (“did not think that treatment might help”, 8.7%), unavailability (8.3%) and treatment stigma (“afraid what people would think about me being in treatment”, 6.7%). Financial and insurance-related barriers were comparatively rare (1.1%). Interestingly, help-seekers more frequently perceived treatment barriers than non-seekers (40.9% vs. 27.8%, *χ*^2^(1) = 9.86, *p* = 0.022). The frequency of specific barriers did not differ in help-seekers vs. non-seekers, and the number of perceived barriers (categorized to one, two, three, more than three) was not associated with help-seeking (*OR* = 1.49, *CI* = 0.91–2.43, *df*_*model*_ = 186). Of the specific barriers, only self-reliance was associated with help-seeking (*OR* = 2.88, *CI* = 1.15–7.20, *df*_*model*_ = 186).

**Table 5:** Frequency of treatment barriers among individuals with AD

	Any AD		PD		AG		SAD		GAD		SPEC	
	N	%	N	%	N	%	N	%	N	%	N	%
Thought about seeking help	365	60.1	77	81.8	114	70.1	74	79.7	60	76.4	218	58.4
Perceived any barrier	187	31.3	33	33.9	53	28.5	42	40.8	31	46.6	114	28.2
Wanted to deal with problem alone	113	18.4	22	24.8	32	15.7	29	25.7	15	23.6	68	17.0
Perceived ineffectiveness	52	8.7	11	14.0	14	6.9	14	13.8	10	11.7	31	7.8
Unavailability	48	8.3	10	10.9	16	9.0	12	12.6	10	15.7	28	6.9
Waiting time too long	27	5.5	7	9.3	10	6.5	7	9.6	5	8.5	15	4.6
Problems with transport/timing	14	2.7	3	5.1	2	1.6	4	5.0	3	5.4	8	2.0
Did not get an appointment	10	2.2	1	2.8	4	1.8	6	7.4	2	3.3	5	2.0
Did not find a provider	12	1.3	3	1.8	4	1.6	3	2.5	2	2.5	9	1.5
Afraid of stigma	34	6.7	7	8.3	12	7.3	13	13.7	6	8.7	18	5.9
Problems with professional	26	4.3	7	7.0	9	6.2	6	5.4	1	2.6	17	4.4
Did not like p.	16	2.7	4	2.5	5	4.1	3	2.1	1	2.6	9	2.1
P. did not take enough time	7	1.4	2	4.3	4	3.0	2	3.2	0	0.0	6	2.1
P. did not see need for treatment	5	0.5	1	0.3	2	0.4	1	0.2	0	0.0	3	0.6
Afraid of hospitalization	11	1.9	3	3.3	5	3.0	5	5.0	0	0.0	8	2.3
Financial	6	1.1	3	4.7	2	0.8	2	3.3	0	0.0	5	1.3

AD = anxiety disorder, PD = panic disorder, AG = agoraphobia, SAD = social anxiety disorder, GAD = generalized anxiety disorder, SPEC = specific phobia

Regarding specific AD, cases with PD most frequently had considered seeking help (81.8%), compared to 58.4% and 34.6% of SPEC without comorbid AD. Treatment barriers were most frequently reported in GAD (46.6%) and least frequently in SPEC (28.2%). Overall, the ranking of different barriers was comparable between diagnoses. By visual inspection, cases with PD more frequently reported perceived ineffectiveness of treatment, while cases with SAD were more frequently deterred by social consequences of seeking treatment (stigma, hospitalization), and cases with GAD less frequently reported self-reliance.

### Treatment Dropout

**Fig. 1:**
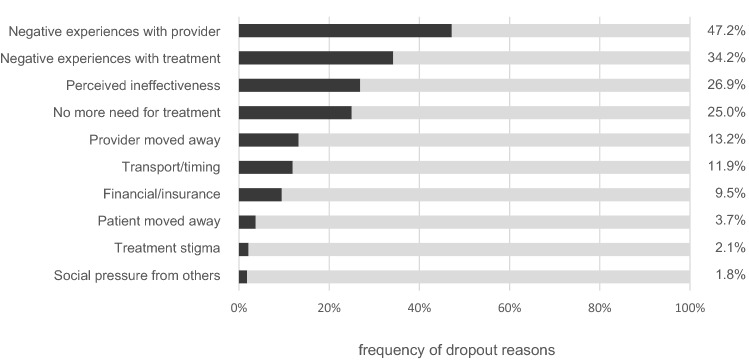
Reasons for dropout among individuals with prior treatment discontinuation (N = 43)

Of the 187 cases with lifetime help-seeking, 27.2% (N = 43) reported discontinuation of at least one mental health treatment. Among those, the most frequent dropout reasons were negative experiences with the provider (i.e., did not get along with provider, 47.1%, or felt mistreated, 11.7%), negative experiences with treatment (i.e. felt out of place, 14.9%, could not manage treatment demands, 12.2%, or negative side effects, 10.6%), and perceived ineffectiveness of treatment (26.9%). Stigma (2.1%) and social pressure from others (1.8%) were rarely a reason for discontinuation (see Fig. 1). Notably, 47.9% of cases with dropout were still receiving disorder-specific interventions during the last 12 months.

## Discussion

Based on a representative community sample of the German adult population, we found that only 26% of all cases with a 12-month diagnosis of AD received any intervention for anxiety problems in their lifetime. In reference to the past 12 months, only 13% received a disorder-specific intervention of any type, irrespective of its adequacy in terms of type, duration and frequency.

### Treatment Barriers

Overall, 40% of the sample never considered seeking help. Moreover, most non-seekers (72%) did not experience barriers to treatment, but simply had never thought about seeking help. The main explicit barriers were self-reliance (wanting to deal with the problem alone; 18%) and assumed ineffectiveness of treatments (9%). A potential explanation of these major barriers might be limited health literacy. Mental health literacy refers to the amount of knowledge and beliefs about mental disorders, including the ability to recognize specific disorders and knowing where to seek help (Jorm et al. 1997). There is evidence for relatively low public health literacy concerning AD. For example, in a case vignette study by Coles et al. (2014) among US adults, less than half of the anxiety vignettes were identified as mental disorders, compared to 62% for a depression vignette, and less than 20% were able to name the AD, compared to more than 50% for depression. Even among psychology students, only a minority were able to identify AD symptoms as indicating a mental disorder (Coles and Coleman 2010). Thus, the inability to recognize anxiety symptoms as mental health problems might be a major reason for not seeking help. Another potential problem is limited knowledge about and/or low trust in the mental health care system. Indeed, there is evidence for lower public trust in healthcare institutions in Germany compared to other western European states (van der Schee et al. 2007) and the moderate satisfaction with treatments in our sample might also point in this direction.

Another relevant barrier was reduced availability of formal help (8%), driven primarily by long waiting periods. The average waiting time for outpatient psychological treatment in Germany is 20 weeks (Federal Psychotherapists’ Chamber 2018) and can be longer for specialized treatments. Long waiting periods reduce the chance to receive treatment in a timely manner and can speak against the use of psychotherapy in acute cases (Bandelow et al. 2014). A third type of relevant barriers was perceived treatment stigma (7%). Stigma has already been found associated with help-seeking, also in AD and specifically in young people (Calear et al. 2020; Clark et al. 2018). Clement et al. (2015) developed a comprehensive model of stigma and help-seeking and conclude that treatment stigma and internalized stigma (shame/embarrassment) are most closely related to help-seeking. Such forms of stigma were not explicitly raised in this study and may underlie some of the prominent “I do not need any help” attitudes in our sample. Noticeably, financial barriers and limited scope of treatment time played a minor role in this population.

Taken together, our findings may indicate that some barriers (financial, basic availability of providers) have already been successfully targeted in the German population, whereas others are still prominent. These are a lack of awareness and, supposedly, mental health literacy for anxiety symptoms and treatments, delayed availability of treatments, and stigma-related barriers.

### Type of Help-Seeking

Among those who crossed the Rubicon to seek formal help for their AD, psychiatrists and psychotherapists were the most frequently used providers. This is encouraging, as psychiatrists and psychotherapists are supposed to be the qualified specialists for diagnostics and treatment of AD. Several points however raise further questions: First, 40% of psychiatric and 20% of psychotherapeutic patients reported only a maximum of five contacts. These patients likely did not get a minimally adequate treatment (Fernández et al. 2007). Second, CBT is the first-line treatment for AD, but only a minority of patients treated by psychotherapists (30%) recall to have received CBT. Third, the majority of cases underwent long-term treatments, whereas treatment studies show that AD are treatable within 20–30 sessions (e.g., Lang et al. 2012), and that major effects are unlikely to occur late in therapy (Cuijpers et al. 2013). Note that the treatment characteristics data must be interpreted with caution. First, only a subsample of n = 92 out of 155 cases answered the interview module on treatment characteristics. Also, this module set rather high demands on participants’ memory and health literacy (what kind of treatment, how many sessions), especially for cases with complex service use histories. Reliability of these data might therefore be limited compared to the rest of the survey. Yet, as detailed information on treatment characteristics are rare in the literature, we chose to subjoin these data.

Remarkably, about half of all patients treated by psychiatrists or psychotherapists were partially or predominantly unsatisfied with their treatment. Dissatisfied patients were also more likely to report treatment dropout. This suggests that in many cases patients’ expectations were not sufficiently met during treatment (Santana and Fontenelle 2011) Further investigations might examine patient expectations as a potential target to prevent dissatisfaction and dropout.

Help-seeking also differed substantially across the AD. In PD, SAD, and GAD, around 40% currently received any kind of disorder-specific intervention, dropping to only 23% in cases with AG and 4% in cases with SPEC. Phobic disorders are thus the most prevalent but also least treated AD, despite efficient and effective treatments available (Zlomke and Davis 2008). Acknowledging that the diagnosis of a mental disorder per se does not imply a need for treatment, this finding is still in sharp contrast to the direct and free access to providers in highly developed mental health care systems such as that in Germany. Diagnosis-specific differences in help-seeking behavior might also reflect the public interest in different disorders. For PD and SAD, large-scale psychotherapy research efforts have been undertaken during the past years (Gloster et al. 2011; Leichsenring et al. 2009), efforts that do not appear to have been made for all AD. Interestingly, the number of used services was highest in SAD, whereas PD and GAD are traditionally seen as conditions with high utilization. This may be explained by the relative intense disability, persistence and overall comorbidity, which, in the German community, seems to be higher in SAD than in other AD.

The help-seeking estimates found here were lower than those reported by others (Bijl and Ravelli 2000; Mack et al. 2014; Mojtabai et al. 2002), but comparable to studies that examined disorder-specific help-seeking (Mackenzie et al. 2012). This reflects that not all cases with any service use also address their anxiety problems. In our sample, this concerns around one third of cases. The gap is largest in SPEC, where only 4% out of 19% with service use also addressed their anxiety, and is virtually nonexistent in PD and GAD (note however, that somatic symptoms of PD and GAD are often misattributed by providers, Wittchen et al. 2002, and see limitations for SPEC). Another reason for the low help-seeking rates could be the use of informal sources, esp. friends and family. Brown et al. (2014) found informal help-seeking to be more prevalent than formal help-seeking in common mental disorders. This is also specifically reported for older individuals, among whom symptoms of AD were not associated with formal help-seeking at all (Hohls et al. 2020).

### Predictors of Help-Seeking

In line with other studies, the strongest predictors of help-seeking were mental-disorder-caused disability and the number of AD (Michel et al. 2018; Reavley et al. 2010; Roness et al. 2005). Strikingly, only 9% of cases with one AD sought help, compared to 68% of cases with three or more AD. This may reflect a tendency to seek help only at a late stage of disorder exacerbation, and may partly explain the high number of long-term psychotherapies. Individuals were also more prone to seek help if they were not in a partnership or were currently unemployed (cf. Roberts et al. 2018). Individuals who are socially integrated may rather rely on informal sources of help (partner, colleagues). The finding that older individuals (65–79 years) were less likely to seek help is consistent with previous findings on AD (Mackenzie et al. 2012), but in contrast with other mental disorders, where lower treatment rates are seen in *younger* adults (Mack et al. 2014; Rickwood et al. 2005). Possibly, anxiety symptoms such as palpitations are often attributed to somatic disorders in older adults, or anxiety symptoms are cared for *en passant* by GPs (Hohls et al. 2019). This finding therefore calls for more rigorous attention to AD in older adults.

### Treatment Dropout

Finally, 28% of help-seekers reported to have already discontinued a mental health treatment, mainly due to negative experiences with providers or applied treatments. This is consistent with the relatively high proportion of dissatisfied patients in our sample. Some patients may enroll in different health services but successfully complete only a few, given the high number of different providers and variety of health services. This interpretation is supported by the fact that 40% of the dropouts were still in treatment by the time of the survey. In sum, dissatisfaction and treatment dropout are frequent among AD patients. Providers should thus be sensitive to thematize prior and current negative experiences during treatment.

### Limitations

Limitations included, first, that we used cross-sectional data, preventing straightforward causal inferences. Second, we examined 12-month AD, whereas individuals with former, now remitted episodes of AD were not included. This resulted in a high mean age of onset (*M* = 36.7, *SD* = 18.6). Third, 12-month use of inpatient services may have been underestimated because hospitalized individuals were not recruited for the survey. Fourth, we relied on participants’ self-report, which is subject to memory bias. For example, some help-seekers may not be aware whether they saw a psychiatrist, psychotherapist, or other medical practitioner. Fifth, 27% of the sample had multiple AD, which may have blurred the distinctions between disorders. Sixth, we did not differentiate primary AD and AD secondary to other problems, which may explain partly why individuals did not seek help due to symptoms of anxiety. Seventh, the item to examine disorder-specific help-seeking for SPEC differed from the other AD (open vs. closed question, see methods) which may have diminished help-seeking percentages in phobias. However, estimates of general service use, that were not biased by the type of question, were also substantially lower in SPEC (Supplement B). Finally, to detect help-seekers we asked participants whether they had *ever* talked to a professional about their AD. It is therefore possible that participants had used mental health services due to their AD before (mean duration of AD was 8.5 years), but only for other reasons in the last 12 months. This may have resulted in overestimated 12-month help-seeking percentages.

### Implications

Our results confirm that help-seeking for AD in Germany is low, despite access to a well-developed healthcare system. Help-seeking seems to be hampered primarily by unawareness, the belief to manage the problems alone and perceived ineffectiveness of treatments. Interventions targeting mental health literacy (Gulliver et al. 2012) are thus needed to inform the public about the symptoms, treatable nature and types of interventions for AD, to decrease stigma and to generate realistic expectations towards mental health and mental health treatments. Large-scale awareness campaigns have been launched for depression and suicidality (Hegerl et al. 2008; Paykel et al. 1998), but not for AD. Other streams of interventions might include training of outpatient therapists and psychiatrists, who carry the brunt of treatment, to apply evidence-based interventions such as exposure (Harned et al. 2014), and the removal of structural barriers perceived by therapists (Pittig et al. 2019). Our results also show that those who seek help do not necessarily talk to their GP, who is commonly supposed to assign appropriate interventions. Thus, providers should continue to engage in active collaboration across service sectors to help patients in finding the most suitable treatment.

## Electronic supplementary material

Below is the link to the electronic supplementary material.

Electronic supplementary material 1 (DOC 66 kb)
