# Supplementary material for: Help-Seeking Behavior and Treatment Barriers in Anxiety Disorders: Results from a Representative German Community Survey
Source: Community Ment Health J. 2021 Jan 20;57(8):1505–17. doi: 10.1007/s10597-020-00767-5 (PMC8531057; doi:10.1007/s10597-020-00767-5)
Supplement: Supplementary file 1 — Electronic supplementary material 1 (DOC 66 kb) [file 10597_2020_767_MOESM1_ESM.doc]

**Supplement A: Item lists from the CIDI’s service use section**

**A.1 Types of service providers**

1. Inpatient health care institutions
   - Psychiatric hospital or department
   - Hospital or department for neurology
   - Psychotherapeutic or psychosomatic hospital
   - Hospital or department for substance abuse
   - Daycare clinic
   - Home for children
   - Other inpatient institutions
2. Outpatient health care institutions
   - General practitioner (if reason was mental health problem)
   - Psychiatrist
   - Psychotherapist
   - Other psychologist
   - Psychiatric or psychotherapeutic outpatient clinic
   - (Social-) psychiatric service
   - Other outpatient institution
3. Complementary institutions
   - Counseling centers
     1. students
     2. education (children, adolescents, parents)
     3. marriage, partnership, life
     4. sexual problems
     5. AIDS support
     6. drug or alcohol use
     7. other counseling centers
   - Self-help organizations
   - Sheltered housing groups
   - Professional education for the disabled
   - Special working places for the disabled
   - Transitional homes
   - Telephone counseling

**A.2 Treatment barriers**

- - Waiting period too long
  - Problems with transport/timing
  - Did not get an appointment
  - Did not find a provider
  - Problems with insurance/finances
  - did not like provider
  - provider did not take enough time for treatment
  - provider did not see need for treatment
  - I wanted to deal with the problem alone
  - I did not think that treatment might help
  - I was afraid what people would think about me being in treatment
  - I was afraid to be hospitalized
  - Other reasons

**A.3 Reasons for treatment dropout**

1. Structural reasons
   - Problems with time or transportation
   - Problems with insurance/payment
   - Treatment was too expensive
2. Provider-related reasons
   - Did not get along with provider
   - Was mistreated
   - Provider moved away
3. Treatment-related reasons
   - Treatment did not help
   - Could not manage treatment demands
   - Negative side effects
4. Internal/psychological reasons
   - Did not need help any longer
   - Felt better
   - felt out of place
   - Afraid of stigmatization
   - Familiy urged me to quit
   - I moved away
5. Other reason

*Note. The items were not grouped into the broader categories A-D in the respondents’ booklet.*

**Supplement B:** Any service use (not specific to anxiety symptoms) in 12-month anxiety disorders

| *Table 2: Any lifetime and 12-month mental health service use in 12-month anxiety disorders (taken from Mack et al., 2014)* | | | | | | | | | | | | |
| --- | --- | --- | --- | --- | --- | --- | --- | --- | --- | --- | --- | --- |
|  | any AD | | PD | | AG | | SAD | | GAD | | SPEC | |
|  | N | %_w_ | N | %_w_ | N | %_w_ | N | %_w_ | N | %_w_ | N | %_w_ |
|  |  |  |  |  |  |  |  |  |  |  |  |  |
| Lifetime service use | 300 | 48.0 | 67 | 72.6 | 98 | 64.0 | 64 | 69.7 | 53 | 69.1 | 175 | 44.1 |
| primary care | 108 | 16.1 | 27 | 30.9 | 42 | 24.9 | 32 | 28.1 | 13 | 10.8 | 66 | 16.4 |
| outpatient services | 230 | 37.9 | 58 | 65.0 | 75 | 48.8 | 55 | 60.4 | 40 | 55.3 | 132 | 34.3 |
| psychotherapist | 151 | 27.2 | 41 | 47.8 | 53 | 39.4 | 42 | 47.5 | 25 | 37.9 | 87 | 24.7 |
| psychiatrist | 104 | 14.8 | 33 | 35.4 | 39 | 22.9 | 28 | 31.5 | 14 | 14.3 | 58 | 13.0 |
| other | 51 | 8.7 | 11 | 13.9 | 18 | 10.4 | 16 | 13.6 | 10 | 13.4 | 29 | 8.6 |
| inpatient services | 109 | 17.4 | 34 | 38.5 | 52 | 33.5 | 31 | 28.4 | 19 | 18.6 | 58 | 14.1 |
| complementary services | 61 | 10.9 | 9 | 14.4 | 23 | 14.9 | 14 | 20.9 | 11 | 16.7 | 35 | 10.2 |
|  |  |  |  |  |  |  |  |  |  |  |  |  |
| 12-month service use | 140 | 23.5 | 36 | 41.8 | 52 | 33.2 | 44 | 48.2 | 32 | 42.6 | 69 | 18.9 |
| primary care | 52 | 7.4 | 15 | 16.6 | 18 | 8.7 | 17 | 12.7 | 9 | 6.8 | 27 | 6.7 |
| outpatient services | 96 | 17.2 | 29 | 35.3 | 37 | 24.4 | 33 | 38.7 | 19 | 28.4 | 50 | 14.1 |
| psychotherapist | 55 | 10.8 | 18 | 21.3 | 22 | 18.3 | 17 | 23.3 | 10 | 16.1 | 28 | 9.1 |
| psychiatrist | 43 | 7.7 | 13 | 18.3 | 18 | 12.6 | 16 | 20.3 | 5 | 5.9 | 25 | 7.3 |
| other | 14 | 2.6 | 5 | 7.2 | 5 | 3.2 | 6 | 5.7 | 5 | 7.0 | 9 | 2.5 |
| inpatient services | 19 | 2.7 | 7 | 7.8 | 11 | 5.8 | 8 | 6.8 | 5 | 4.6 | 9 | 1.9 |
| complementary services | 17 | 4.2 | 3 | 6.8 | 7 | 6.1 | 4 | 7.7 | 3 | 9.8 | 10 | 3.8 |
| *Note:* AD = anxiety disorder, PD = panic disorder, AG = agoraphobia, SAD= social anxiety disorder, GAD = generalized anxiety disorder, SPEC = specific phobia | | | | | | | | | | | | |
